# D-dencichine Regulates Thrombopoiesis by Promoting Megakaryocyte Adhesion, Migration and Proplatelet Formation

**DOI:** 10.3389/fphar.2018.00297

**Published:** 2018-04-03

**Authors:** Shilan Ding, Min Wang, Song Fang, Huibo Xu, Huiting Fan, Yu Tian, Yadong Zhai, Shan Lu, Xin Qi, Fei Wei, Guibo Sun, Xiaobo Sun

**Affiliations:** ^1^Beijing Key Laboratory of Innovative Drug Discovery of Traditional Chinese Medicine (Natural Medicine) and Translational Medicine, Institute of Medicinal Plant Development, Peking Union Medical College and Chinese Academy of Medical Sciences, Beijing, China; ^2^Key Laboratory of Bioactive Substances and Resource Utilization of Chinese Herbal Medicine, Ministry of Education, Beijing, China; ^3^Zhongguancun Open Laboratory of the Research and Development of Natural Medicine and Health Products, Beijing, China; ^4^Key Laboratory of Efficacy Evaluation of Chinese Medicine Against Glycolipid Metabolic Disorders, State Administration of Traditional Chinese Medicine, Beijing, China; ^5^Kunming Shenghuo Pharmaceutical Group Co., Ltd., Kunming, China; ^6^Academy of Chinese Medical Sciences of Jilin Province, Jilin, China; ^7^Department of Oncology, Guang’anmen Hospital, China Academy of Chinese Medical Sciences, Beijing, China

**Keywords:** chemotherapy-induced thrombocytopenia, thrombopoietin, D-dencichine, platelets, cytokines, mouse

## Abstract

Life-threatening chemotherapy-induced thrombocytopenia can increase the risk of bleeding due to a dramatic low platelet count, which may limit or delay treatment schedules in cancer patients. The pressing need for the rapid alleviation of the symptoms of thrombocytopenia has prompted us to search for novel highly effective and safe thrombopoietic agents. Pharmacological investigations have indicated that dencichine can prevent and treat blood loss and increase the number of platelets. On the basis of the neurotoxicity of dencichine, D-dencichine is artificially synthesized in the laboratory. Our initial results showed that D-dencichine had potential to elevate peripheral platelet levels in mice with carboplatin-induced thrombocytopenia. However, the mechanisms of D-dencichine on thrombopoiesis have been poorly understood. In this study, we found that sequential administration of D-dencichine had a distinct ability to elevate numbers of reticulated platelets, and did not alter their clearance. Moreover, we demonstrated that D-dencichine was able to modulate the return of hematopoietic factors to normal levels, including thrombopoietin and IL-6. However, subsequent analysis revealed that D-dencichine treatment had no direct effects on megakaryocytes proliferation, differentiation, and polyploidization. Further *in vitro* studies, we demonstrated for the first time that D-dencichine significantly stimulated megakaryocyte adhesion, migration, and proplatelet formation in a dose-dependent manner through extracellular regulated protein kinases1/2 (ERK1/2) and v-akt murine thymoma viral oncogene homolog (AKT) signaling pathways. This study sufficiently characterized the role of the effects of D-dencichine treatment on the regulation of thrombopoiesis and provided a promising avenue for CIT treating.

## Introduction

Platelets play essential roles in repairing vascular damage, initiating thrombus formation in the event of overt vascular injury, repairing wound, regulating innate immune response, and metastatic tumor cell biology (Tantry et al., 2010; Jenne et al., 2013). They are produced daily from bone marrow (BM) megakaryocytes, which must be differentiated from hematopoietic stem cells (HSCs) by undergoing several discrete stages to increase the committed progenitors (Tantry et al., 2010). Under the regulation of factors, such as thrombopoietin (TPO), chemokines, or the localized expression of ligands for megakaryocyte surface receptors, early megakaryocytes undergo a proliferative stage to increase their numbers; afterward, they are followed by multiple rounds of endomitosis where the diploid promegakaryocytes undertake DNA duplication without cell division to accumulate a DNA content of 4 N up to 128 N (Ravid et al., 2002). Megakaryocytes differentiation is predominantly driven by the TPO-dependent signaling *in vitro* and *in vivo*. TPO binding to its receptor, c-Mpl, results in the activation of Janus kinase 2 (JAK2). JAK2 phosphorylates tyrosine residues on c-Mpl, which prompts activation of its downstream signaling pathways, such as signal transducer and activator of transcriptions 3/5 (STAT3/5) and other pathways. Additionally, ERK1/2 and Akt are two important positive regulators of the TPO-dependent signaling (Mazharian et al., 2009). Once mature, megakaryocytes must migrate within the complex BM stromal environment from an osteoblastic niche to a vascular niche, which is the site of platelet production. Mature megakaryocytes can convert their cytoplasm with a cytoskeleton-driven process into long protrusions (proplatelets); then the final platelets are shaped (Patel et al., 2005; Bender et al., 2014).

Chemotherapy-induced thrombocytopenia (CIT), although less frequent than granulocytopenia in clinical, may represent a life-threatening and less easily controlled event in cancer patients. Besides the bleeding risk, CIT could lead to delay or limit in the chemotherapy schedules, which are associated with poorer outcomes in the treatment of patients who have received chemotherapy. In clinical medication, some well-known anti-cancer drugs inducing apoptosis of immature hematopoietic cells is the major cause of thrombocytopenia in cancer patients, such as carboplatin, cisplatin, cytosine arabinoside, oxaliplatin, vincristine (Ulich et al., 1995). Platelet transfusion is administered to prevent or treat thrombocytopenia when patients applied in high-dose settings. This standard treatment for thrombocytopenia is associated with several risks, such as alloimmunization, infection transmission, transfusion reactions, and platelet refractoriness (Vadhan-Raj, 2009). In recent years, several pleiotropic hematopoietic growth factors and cytokines with thrombopoietic activity have been considered to play a regulatory role in thrombopoiesis, such as IL-3, TPO, pegacaristim, promegapoietin, and peptide agonists capable of binding and activating the TPO receptor. The main disadvantage of these drugs is an overall long time lapse before peak platelet response (Jelic and Radulovic, 2006). Furthermore, the development of recombinant protein drugs derived from TPO for the treatment of thrombocytopenia has been forced to stop because of the undesired immunogenicity of the drugs. The failure to develop TPO-derived drugs has forced us to search for new reagents to treat thrombocytopenia. At present, IL-11 is the only cytokine licensed in the United States for the treatment of CIT, but its thrombopoietic activity is modest and its use is often associated with unfavorable side effects (Zeuner et al., 2007). Therefore, novel thrombopoietic agents should be developed, and natural products from Traditional Chinese Medicine have become important resources of novel thrombopoietic agents.

**FIGURE 1:**
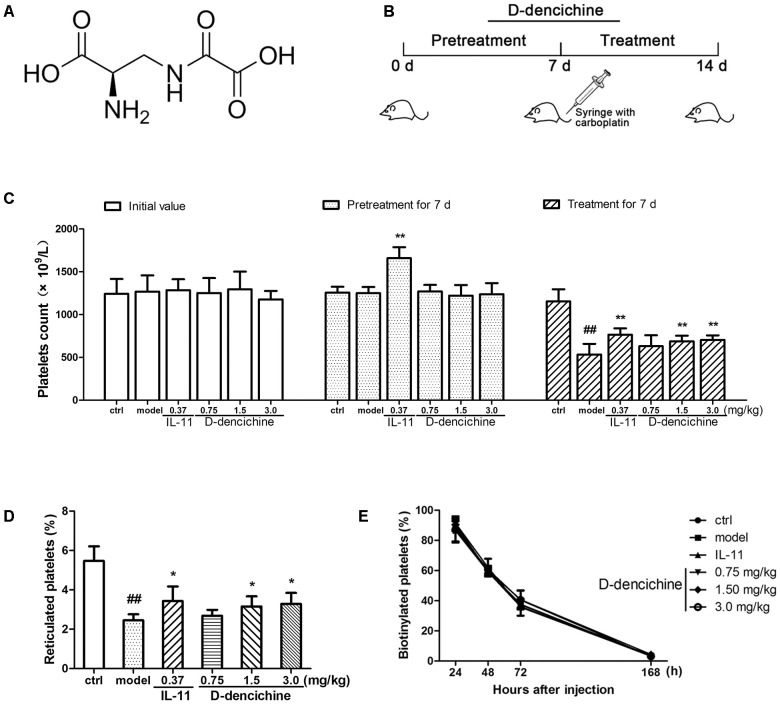
D-dencichine treatment increases the number of circulating platelets in mice with thrombocytopenia induced by carboplatin. **(A)** The chemical structure of D-dencichine. **(B)** Experimental schedule of D-dencichine administration in Balb/c mice. The six group mice were received NS or IL-11 or D-dencichine for 7 days. On the 7th day, the other five groups then intravenously injected with carboplatin (80 mg/kg), whereas the control group was intravenously injected with volumetric NS. After that, each group was treated with NS or IL-11 or D-dencichine for another 7 days. **(C)** Peripheral platelet counts under the administration of IL-11 (0.37 mg/kg) or D-dencichine (0.75, 1.50, and 3.0 mg/kg) in mice for different time intervals (0, 7, and 14 days) subjected to thrombocytopenia induced by carboplatin (on day 7), (*n* = 10). **(D)** Reticulated platelet count was determined by flow cytometry. **(E)** Quantification of the percentage of platelets *in vivo* biotinylated platelets 24, 48, 72, and 168 h after sulfo-NHS-biotin injection (1 h after injection; *n* = 5). ^##^*P* < 0.01 vs. control group; ^∗^*P* < 0.05, ^∗∗^*P* < 0.01 vs. model group.

*Panax notoginseng* (Burk) F. H. Chen, namely Tianqi or Sanqi, is a well-known Traditional Chinese Medicine in China and Japan. It has been selected as a therapeutic drug for treatment of blood disorders, including blood stasis, bleeding, and blood deficiency. The steamed form of *P. notoginseng* has been claimed to be a tonic used to “nourish” blood and to increase production of various blood cells in anemic conditions (Lau et al., 2009). Dencichine, or β-N-oxalyl-L-a, β-diaminopropionic acid (β-L-ODAP), is a non-protein amino acid that was originally extracted from the roots of *P. notoginseng*. Pharmacological investigations have indicated that dencichine can prevent and treat blood loss and increase the number of platelets (Jie et al., 2017). However, multiple investigations of dencichine-induced lathyrism demonstrated that it has neurotoxic potential (Pratap Rudra et al., 2004; Xu et al., 2017). Therefore, D-dencichine is synthesized on the bases of the chemical structure of dencichine, and its molecular structure was shown in **Figure 1A**. Our initial results showed that D-dencichine increased platelet counts in mice with thrombocytopenia induced by carboplatin, and the effects were equivalent to dencichine. However, the underlying mechanisms of D-dencichine on thrombopoiesis have not yet been elucidated.

The purpose of the present study is to report the ability of D-dencichine to significantly ameliorate carboplatin-induced thrombocytopenia and to determine its action mechanisms. We found that D-dencichine was efficient in promoting megakaryocyte adhesion, migration, and proplatelet formation (PPF) through ERK1/2 and Akt signaling pathways. These effects lead to an increase in the platelet production and may stimulate platelet recovery in thrombocytopenic mice induced by chemotherapy.

## Materials and Methods

### Reagents

The target product D-dencichine (98% purity) was from the Institute of Medicinal Plant Development, Peking Union Medical College and Chinese Academy of Medical Sciences (Beijing, China). Recombinant human (rh) IL-11 was purchased from Qilu Pharmaceutical Co., Ltd. (Jinan, China). Recombinant murine (rm) TPO and rhTPO were obtained from PeproTech Inc. (Rocky Hill, NJ, United States). Stem cell factor (rhSCF) was from R&D Systems (Minneapolis, MN, United States). Primary monoclonal antibodies against phospho-JAK2 (Y1007+Y1008), JAK2, phospho-STAT5 (Y694), STAT5α+STAT5b, phospho-STAT3 (Y705), STAT3, phospho-Akt (S473), Akt, c-Mpl, and Bcl-2 were purchased from Abcam (Cambridge, MA, United States). Primary monoclonal antibodies against phospho-ERK1/2, ERK1/2, Bax polyclonal primary antibody, goat anti-rabbit horseradish peroxidase (HRP) and rabbit anti-mouse HRP were obtained from Santa Cruz Biotechnology (Santa Cruz, CA, United States). APC-labeled anti-mouse CD41 was from eBioscience (San Diego, CA, United States). FITC-labeled anti-human CD41α, APC-labeled anti-human CD42b, PI/RNase staining buffer were purchased from BD Biosciences (La Jolla, CA, United States). The cell culture materials were acquired from Gibco (Grand Island, NY, United States). Plasma fibronectin and fluorescent phallotoxins were from Invitrogen (Eugene, OR, United States). All reagents were purchased from Sigma-Aldrich (St. Louis, MO, United States) unless otherwise stated.

### Mice and Experimental Details

Healthy male Balb/c mice (7 or 8 weeks old and 18–22 g in weight) were purchased from Vital River Laboratory Animal Technology Co., Ltd. (Beijing, China). The mice were maintained under a 12 h/12 h light/dark cycle and given free access to sterilized food and purified water for 1 week in room temperature (RT) at 25 ± 1°C and humidity of 60%. The mice were randomly divided into six groups. (1) The mice in the control group were injected intraperitoneally with normal saline (NS). (2) The mice in the model group were treated with NS for 7 days and injected intravenously with 80 mg/kg carboplatin on day 7. (3) The mice in the positive group were injected subcutaneously with 0.37 mg/kg IL-11. (4) The mice in D-dencichine treatment groups were intraperitoneally injected with 0.75, 1.5, and 3.0 mg/kg D-dencichine. As shown in **Figure 1B**, these mice in the experimental groups received NS, IL-11, or D-dencichine for 7 days and then intravenously injected with carboplatin, whereas the control group was intravenously injected with volumetric NS. Each group was given the same treatments for another 7 days. After the 14-day treatment, the mice were deprived of food overnight and euthanized. All of the animal experiments were approved by the Research Ethics Committee of the Chinese Academy of Medical Sciences and Peking Union Medical College, Beijing, China (SCXK 2014-0001).

### Cell Culture

Murine fetal livers from 14.5 days postcoital were prepared into single-cell suspension by successively passing through 18- and 22-gauge needles and filtering with a 70 μm cell strainer. The cells were cultured in Dulbecco modified Eagle medium (Gibco Life Technologies, United States) supplemented with 10% FBS, 100 U/mL penicillin, 100 μg/mL streptomycin, and 50 ng/mL rmTPO for 5 days. Changing the medium or supplementing with additional rmTPO during the culture period was unnecessary.

### Measurement of Hematologic Parameters

Platelets in the peripheral blood from the retro-orbital plexus of the mice were bled through heparinized capillaries into EDTA-coated tubes; the samples were collected on the indicated days and counted automatically in a hematology analyzer (Sysmex XT-1800i/2000IV, Kobe, Japan). Hematopoietic factors in serum samples were determined using the commercially available murine TPO quantikine ELISA kit (R&D Systems, Minneapolis, MN, United States) and murine IL-6 ELISA kit (Abcam, Cambridge, MA, United States) according to the manufacturer’s protocol.

### Platelet Production Assay

To assess platelet production (reticulated platelets) *in vivo*, a small amount of whole blood was collected from tail vein and placed in anticoagulant. One hundred microliter thiazole orange (TO, 0.1 μg/mL in PBS), 18 μL antibody solution (CD41-APC 1/40 in PBS), and 2 μL blood were mixed. The mixture was pipetted gently and incubated for 30 min at RT. Light exposure was avoided. Each sample was fixed in 1% paraformaldehyde for 15 min and analyzed immediately on a FACSCalibur (BD Biosciences, San Jose, CA, United States). TO-positive platelets were considered reticulated (Prislovsky et al., 2008).

### Platelet Clearance Analysis

*In vivo* biotinylation approach (double labeling platelets) was used to determine platelet clearance as reported in the literature (Prislovsky et al., 2008). Briefly, mice were injected via tail vein with 35 μg/g body weight sulfo-NHS-biotin (BioVision, United States). The blood was collected from the tail vein at the indicated time points and placed directly in microfuge tubes. The blood samples were then diluted 20× in anticoagulant mix, and centrifuged at 125 × *g* at RT. One hundred microliter supernatant was collected. CD41-APC antibody and streptavidin-PE (BD Biosciences) were added to label the biotinylated platelets for 40 min at RT in the dark. After fixation in 1% paraformaldehyde, the samples were analyzed via flow cytometry.

### Megakaryocyte Polyploidy Analysis

Murine femoral and tibia were dissected out and the marrow was flushed out with buffer solution. Mouse spleen capsule was cut at one end, and the cells were gently squeezed out from the cut end using blunt forceps. A single-cell suspension was prepared with three or four passages through a 3-ml syringe and 23-gauge needle, and red cells were lysed on ice using the lysate buffer (Beyotime, China). For the baseline ploidy analysis, cells were analyzed as described previously with minor modifications (Fuhrken et al., 2008). BM and spleen cells were harvested and labeled with an APC-conjugated CD41 antibody for 30 min at 4°C. The cells were then washed twice in 2 mM EDTA in PBS and fixed in 0.5% paraformaldehyde for 10 min at RT. The fixed cells were permeabilized with 70% methanol for 1 h at 4°C, then stained with propidium iodide (PI) solution (0.5 mL/test) containing RNase for 15 min at RT, and analyzed through flow cytometry.

### Human Primary Megakaryocytes Analysis

Hematopoietic stem and progenitor Cells (CD34^+^) were purchased from Nuo Wei Biotechnology Co., Ltd. (Beijing, China). The purity of CD34^+^ cells is >90% by flow cytometry. CD34^+^ cells were cultured in serum-free medium (StemSpan SFEM, Stem Cell Technologies, Canada) supplemented with different growth factors, including 20 ng/mL rhSCF and 10 ng/ml rhTPO. Cells were seeded in 24-well plates at a density of 4 × 10^4^/mL, and were cultured for different days. A total of 1 × 10^5^ cultured cells were collected at the indicated time points, and were labeled with FITC-conjugated mouse anti-human CD41α and APC-conjugated mouse anti-human CD42b antibodies for 30 min at RT in the dark. The cells were then fixed in 1% paraformaldehyde and analyzed through flow cytometry.

### Cell Viability Assay

The M07e and Meg-01 cells were purchased from the American Type Culture Collection (Manassa, VA, United States). The cells were cultured in Roswell Park Memorial Institute (RPMI) 1640 medium (Hyclone, United States) containing 10% heat-inactivated FBS and kept in a humidified incubator with 5% CO_2_ at 37°C. These cells were seeded into 96-well plates at a density of 5 × 10^4^/well in the presence of various D-dencichine concentrations and grown for 24, 48, and 72 h. Ten microliter CCK-8 regent (Dojindo, Japan) was then added to each well and incubated for 5 h. The absorbance was measured on a microplate reader at 450 nm (Infinite M1000, Tecan, Sunrise, Austria).

### c-Mpl Expression Determined With Flow Cytometry

Bone marrow cells and platelets were stained with a biotinylated rat anti-mouse c-Mpl mAb (clone AMM2, Immuno-Biological Laboratories, Minneapolis, MN, United States) for 30 min, followed by streptavidin-APC (BD Biosciences) for 40 min, and was determined by flow cytometry.

### Histology Analysis

Dissected tissues (sternum, liver and spleen) from four mice selected randomly from each group were removed and fixed overnight in 10% formaldehyde. Sternum was decalcified in Morse’s solution (10% sodium citrate and 22.5% formic acid) after fixing. Specimens were embedded in paraffin, cut into 5 μm thick sections, and stained with hematoxylin and eosin (H&E) using standard methods. Then we took photos under a microscope and the numbers of megakaryocytes in three microscopy fields per slide were counted under the microscope.

### Western Blot Analysis

Platelet pellets were prepared as previously described with minor modifications (Lebois et al., 2016). Platelet-rich plasma was obtained by centrifugation at 125 × *g* for 8 min, followed by centrifugation of the supernatant buffy coat at 125 × *g* for 8 min. Platelets were washed by two sequential centrifugations at 860 × *g* in specific buffer. Western blotting analysis was performed as described (Yu et al., 2014). Cells were lysed in cold lysis buffer (Solarbio, China), and proteins were separated using sodium dodecyl sulfate polyacrylamide gel electrophoresis and transferred onto a nitrocellulose membrane (Bio-Rad, United States). The membranes were washed, blocked, and incubated with the primary antibody and with an appropriate HRP-conjugated secondary antibody afterward. Images were scanned using Image Lab software (Bio-Rad, Hercules, CA, United States) and the relative density of immunoreactive bands was determined using the Quantity One software.

### Adhesion Assay

Meg-01 cells were loaded with 5 μM BCECF-AM (Beyotime, China) for 1 h at 37°C. The labeled cells (1 × 10^5^ cells/well) were seeded in a 24-well culture plate precoated with 100 μg/mL fibrinogen and cultured for 3 h at 37°C in serum-free RPMI 1640 medium supplemented with or without D-dencichine. The cells were then washed three times with PBS, and the fluorescence of adhesive cells was determined on a microplate reader.

### Transwell Migration Assay

To assess megakaryocyte migration, 24-transwell chambers with 8-mm pore size polycarbonate membranes (Corning, United States) were coated with 20 μg/mL fibronectin overnight at 4°C. Meg-01 cells were serum starved overnight, and 2 × 10^5^ cells were placed in the upper chamber containing serum-free RPMI 1640 medium with 0.2% bovine serum albumin (BSA). The lower chamber comprised serum-free RPMI 1640 medium with or without D-dencichine (25, 50, and 100 μM). The cells were allowed to migrate for 5 h at 37°C, and the unmigrated cells were removed. The membrane was stained with crystal violet (Beyotime, China) to visualize the cells. The number of migrated cells was examined under an inverted microscope (Molecular Devices, United States). The dye on the membrane was eluted with 33% acetic acid, and crystal violet absorbance was measured with a microplate reader at 570 nm.

### PPF Assay

Proplatelet formation was performed as previously described with minor modifications (Larson, 2006). Briefly, the fetal liver-derived primary megakaryocyte cells were expanded with 50 ng/mL rmTPO for 5 days and purified over a discontinuous BSA density gradient (1.5%/3%). The purified megakaryocytes were seeded in 200 μg/mL fibrinogen-precoated plates for 5 h at 37°C. The cells were fixed with 10% formalin, permeabilized with 0.25% Triton X-100, and stained with fluorescent phallotoxins, the anti-β1-tubulin antibody and DAPI (Beyotime, China). Images were captured using a fluorescence microscope, and the number of megakaryocyte-producing proplatelets was enumerated.

### Statistical Analysis

Experimental data in this report are expressed as the mean + standard deviations (SDs) of at least three independent experiments. Statistical analyses were performed by Student’s *t*-test to assess the differences between two groups. Comparisons among the multiple groups were performed using the two-way analysis of variance (ANOVA). *P* < 0.05 was considered statistically significant.

## Results

### D-dencichine Treatment Increases the Number of Circulating Platelets in Mice With Thrombocytopenia Induced by Carboplatin

Carboplatin is a second generation platinum drug that is highly effective in the treatment of malignant tumors. Among carboplatin’s toxicities are myelosuppression and thrombocytopenia (Ulich et al., 1995). Therefore, a mouse model of thrombocytopenia induced by carboplatin was applied in this study. To examine whether D-dencichine treatment could ameliorate CIT, we pretreated the randomized mice with different D-dencichine doses (0.75, 1.5, and 3.0 mg/kg) for 7 days and used rhIL-11 as a positive control. When the platelets in a whole blood sample were enumerated using a hematology analyzer on day 7, we observed that D-dencichine could not elevate the platelet level in the peripheral blood, whereas rhIL-11 increased the platelet counts by 29% more than that of the control group (**Figure 1C**). Carboplatin was then intravenously injected to the mice to induce severe thrombocytopenia on day 7. D-dencichine and rhIL-11 were administered for another 7 days after carboplatin stimulated myelosuppression. Both D-dencichine and rhIL-11 were effective in promoting platelet counts compared with the model control. The platelet counts in the D-dencichine treatment mice (0.75, 1.5, and 3.0 mg/kg) and the rhIL-11 group were approximately 18, 29, 32, and 44% compared with that in the model group, respectively. Differences between the D-dencichine treatment group (1.5 and 3.0 mg/kg) and the model group were statistically significant. The results indicated that D-dencichine had a capacity to heighten the platelets counts of mice suffering from thrombocytopenia induced by carboplatin.

The increased platelet number in mice could arise from increased production or decreased clearance. To clarify the causes of increased platelet counts with D-dencichine treatment, we first detected platelet production by TO staining because it specifically combines with RNA in newly formed platelets (reticulated platelets). As shown in **Figure 1D**, an increase of newly formed platelets in D-dencichine treatment groups was observed compared with that in the model group. To further investigation the effects of D-dencichine-heightened platelet number, we then assessed the platelet clearance in groups using an *in vivo* biotinylation method. In contrast, the percentage of platelets cleared was unaltered each group (**Figure 1E**). These data suggested that the increased platelet count with D-dencichine treatment probably resulted from promoted platelet production but not platelet clearance.

### D-dencichine Played a Regulatory Role in Control of Hematopoietic Factors

**FIGURE 2:**
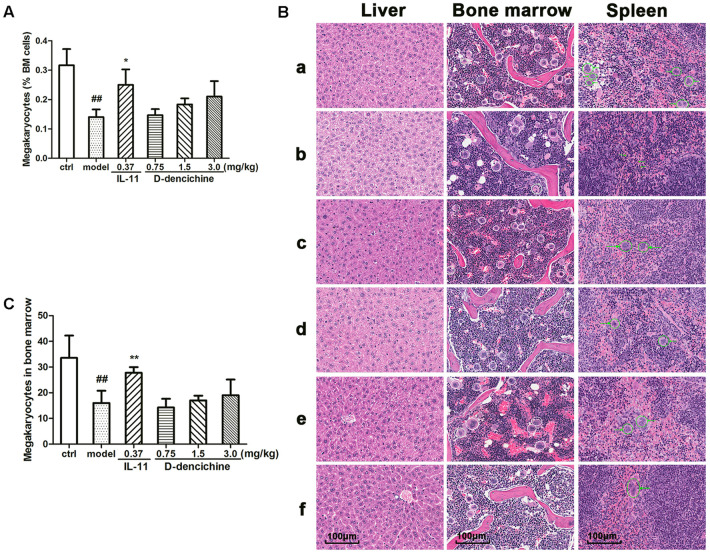
Effects of D-dencichine on carboplatin-induced thrombocytopenia *in vivo*. Mice were treated with NS or rhIL-11 or D-dencichine. **(A)** The numbers of megakaryocytes in BM were determined using flow cytometry. **(B)** Effects of D-dencichine treatment on histological changes in liver, bone marrow, and spleen in mice by H&E staining (200×). a: control group; b: model group; c: positive group; d–f: D-dencichine (0.75, 1.50, and 3.0 mg/kg) groups. White circles mark the megakaryocytes in mice bone marrow, while green circles label the megakaryocytes in mice spleen. The green arrows in the model group show the bleeding points. **(C)** The numbers of megakaryocytes in BM were counted from the information in **(B)**. ^##^*P* < 0.01 vs. control group; ^∗^*P* < 0.05, ^∗∗^*P* < 0.01 vs. model group.

Platelets are released from megakaryocytes residing in BM, which has been proven as a major site of platelet production in mice. Using flow cytometry, we found that D-dencichine treatment group had no more megakaryocytes per nucleated cells in BM than in the model group (**Figure 2A**). In addition to the BM, the liver and spleen are also important sites of hematopoiesis in mice. Therefore, we evaluated the pathological changes in liver, spleen, and sternum to verify the role of D-dencichine in thrombopoiesis facilitation *in vivo*. H&E staining revealed that the numbers of megakaryocytes in the BM of D-dencichine treatment group did not increase compared with that in the model group (**Figures 2B,C**), which further supported the aforementioned observation. Through histologic observations, the spleen megakaryocyte numbers were lower in the model group compared with the other groups, but getting an accurate megakaryocyte counts was not easy because the distribution of megakaryocytes was particularly uneven in spleen. In contrast to the BM and spleen, the liver in the model group was infiltrated by focal necrosis and inflammatory cells. The liver cells of D-dencichine treatment groups were swollen, but the lesions were relieved. These results suggested that D-dencichine treatment promoting thrombopoiesis *in vivo* must be caused by other mechanisms.

**FIGURE 3:**
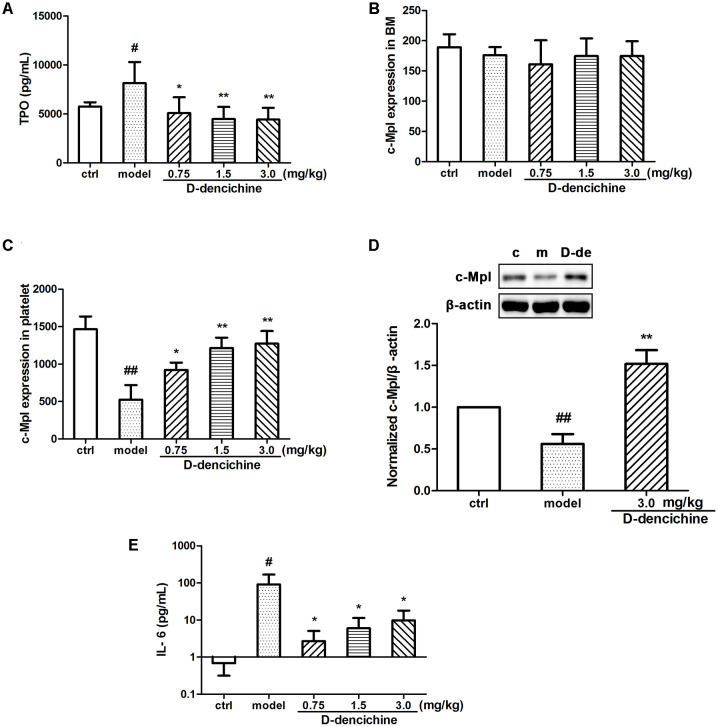
The regulation effects of D-dencichine on hematopoietic factors. **(A)** The level of thrombopoietin (TPO) was measured by ELISA. **(B,C)** The c-Mpl expression level in BM and platelets were analyzed by flow cytometry. **(D)** The protein expression level of c-Mpl was determined by western blot and quantified using densitometric analysis. **(E)** The concentration of IL-6 was determined by ELISA. ^#^*P* < 0.05, ^##^*P* < 0.01 vs. control group; ^∗^*P* < 0.05, ^∗∗^*P* < 0.01 vs. model group.

It is known that the process of megakaryocyte maturation is driven by several factors and chemokines. TPO is the primary regulator of platelet production and supports survival, proliferation, and differentiation of megakaryocytes in BM (Szalai et al., 2006; Kaushansky, 2009; Kuter, 2009). We hypothesized that the elevated platelet counts in mice because of the increased serum TPO lever in the D-dencichine treatment group. To confirm this speculation, we tested serum TPO concentrations in groups. Surprisingly, serum TPO levers in D-dencichine treatment groups were lower than those in the model group (**Figure 3A**). TPO acts by binding itself to a specific cell surface receptor (c-Mpl), which is required for rapid response to platelet loss. Therefore, we analyzed the surface expression of c-Mpl protein in BM cells and platelets through flow cytometry. We found that the c-Mpl expression in platelets of D-dencichine treatment groups increased compared with that of the model group (**Figure 3C**). Furthermore, western blot analysis of c-Mpl protein expression in platelets increased in D-dencichine treatment group at a dose of 3.0 mg/kg, which further supported the results (**Figure 3D**). However, c-Mpl expression in BM cells using flow cytometric analysis was unaltered (**Figure 3B**). We concluded that circulating TPO concentrations might be inversely proportional to the “c-Mpl mass,” which was contributed by platelet counts. Therefore, our speculation about the possibility that altered expression levels of TPO and c-Mpl being directly responsible for the increased platelets could be ruled out. Megakaryopoiesis and platelet production are tightly regulated by additional growth factors and cytokines to maintain a normal number of circulating platelets, such as IL-3, SCF, IL-6, and IL-11. Among these growth factors, IL-6 has been shown to act directly on megakaryocytes to increase platelet production. To learn more about how D-dencichine affects IL-6 expression in mice, we tested serum IL-6 concentrations. Correspondingly, IL-6 concentrations in D-dencichine treatment group were lower compared with that in the model group in a manner that was similar to the effect of TPO (**Figure 3E**). The data suggested that IL-6 concentrations in the model group were high and its concentrations in the D-dencichine treatment group were in the low lever, which must have been caused by another mechanism.

### D-dencichine Has No Direct Effects on Megakaryocyte Proliferation, Differentiation, and Polyploidy

**FIGURE 4:**
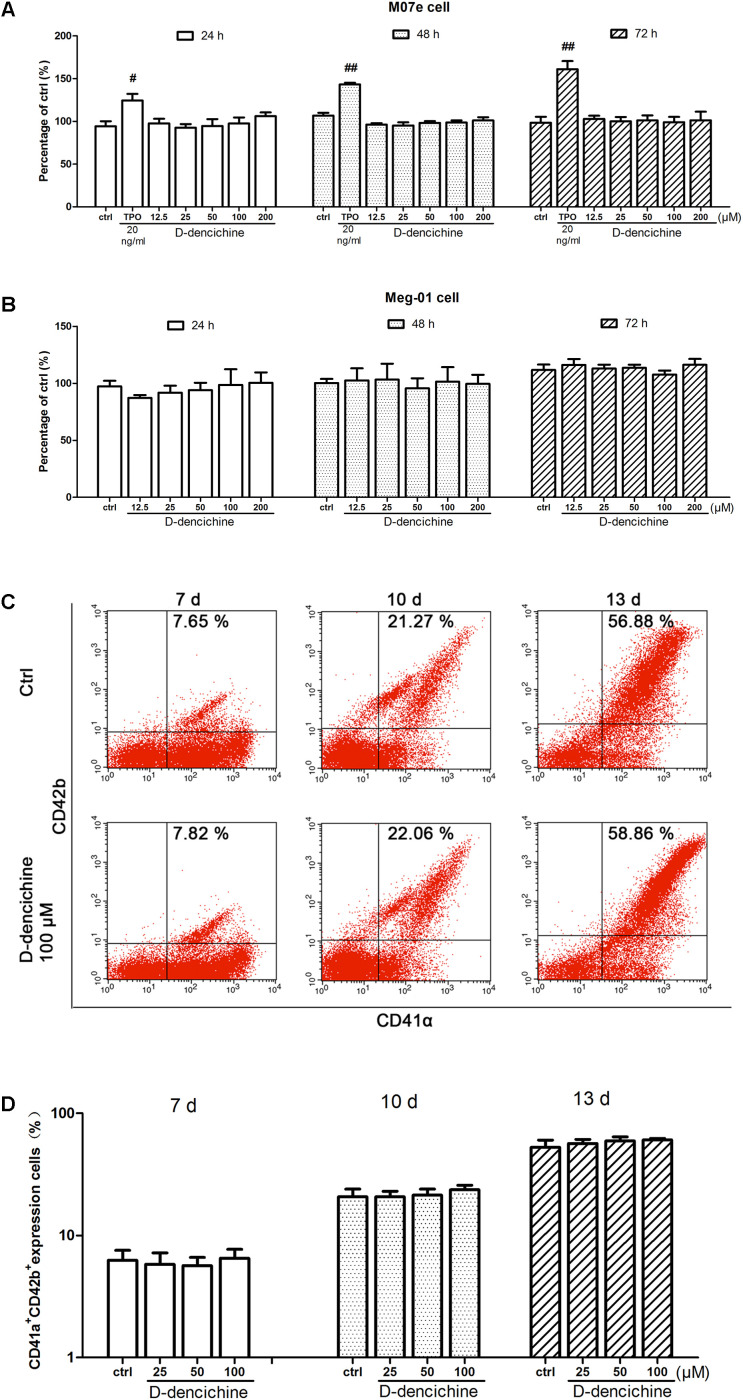
D-dencichine has no direct effects on the proliferation and differentiation of megakaryocytes *in vitro*. **(A,B)** Viability of M07e and Meg-01 cells cultured with D-dencichine (12.5, 25, 50, 100, and 200 μM) for 24, 48, and 72 h as measured by CCK-8 assay. The data are from six independent assays with a single batch of cells. **(C)** Human cord blood-derived CD34^+^ cells were cultured with or without different concentrations of D-dencichine (25, 50, and 100 μM) together with rhSCF (20 ng/ml) and rhTPO (10 ng/ml) for 7, 10, and 13 days. The expressions of CD41α and CD42b in the cells treated with D-dencichine were analyzed through flow cytometry. **(D)** Histogram showing the percentage of CD41α^+^CD42b^+^ megakaryocytes for each group from the information in **(C)**. ^#^*P* < 0.05, ^##^*P* < 0.01 vs. control group.

**FIGURE 5:**
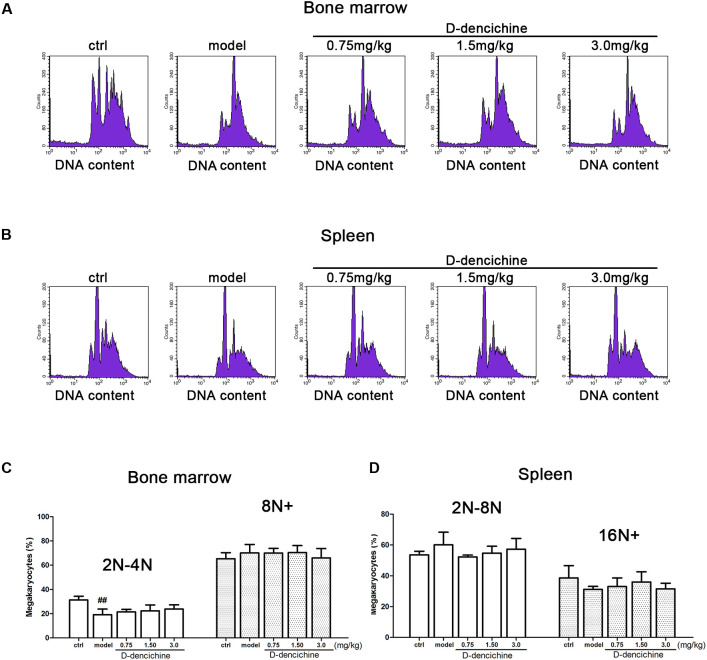
D-dencichine was unable to promote megakaryocytic polyploidization. **(A,B)** The distribution of DNA contents of primary megakaryocytes in mouse BM and spleen through flow cytometry, by means of double-labeled with APC-conjugated CD41 and PI. **(C,D)** Histogram showing the DNA content in CD41^+^ cells in mouse BM and spleen. ^##^*P* < 0.01 vs. control group.

We performed a series of experiments *in vitro* to investigate the direct effects of D-dencichine treatment on megakaryocytes proliferation and/or differentiation. First, we chose the megakaryoblastic cell line M07e, the mature megakaryocyte cell line Meg-01, and human primary megakaryocytes derived from umbilical cord blood CD34^+^ cells that were cultured with or without D-dencichine at different doses (25, 50, and 100 μM) for different days. As shown in **Figures 4A,B**, we found that D-dencichine treatment could not promote the proliferation in both M07e and Meg-01 cells. During development, megakaryocytes undergo a series of transformations that can be identified by expression specific surface proteins, including GPIIb (also known as the integrin subunit aIIb or CD41) and GPIb (GPIb-V-IX complex, CD42b), in association with nuclear maturation and subsequent cytoplasmic maturation (Mazharian et al., 2009). Megakaryocytes development starts expressing integrin CD41α and maturation marker CD42b on their surface upon differentiation from HSCs. The well-known megakaryocyte cell lines, such as M07e, Meg-01, and K562, produced mostly CD41α^+^, but CD42b^-^ (Nakamura et al., 2014). To detect whether D-dencichine has a direct effect on megakaryocyte differentiation, cord blood-derived CD34^+^ cells were cultured with or without different doses of D-dencichine supplemented by adding rhSCF and rhTPO for 7, 10, and 13 days. Afterwards, we detected the surface expression levels of CD41α^+^CD42b^+^ via flow cytometry. However, our data showed that D-dencichine had no significant effect on megakaryocyte differentiation (**Figures 4C,D**). Megakaryopoiesis is an especial process that involves gradual differentiation of immature megakaryocyte progenitors into diploid megakaryocytes, which undergoes a progressive megakaryocytic polyploidization and a subsequent process of cytoplasmic maturation leading to platelet release. Platelet production was considered to correlate positively with megakaryocyte polyploidy (several rounds of chromosomal duplication without cell division) to increase DNA contents. Therefore, we determined whether BM and spleen megakaryocytes DNA contents were altered with D-dencichine treatment using flow cytometry. Particularly, we found that DNA contents (2N-4N) of immature megakaryocyte progenitors in model group were significantly lower compared to the control group, but they were not altered in spleen cells. The observed phenomenon might be related to the ability of carboplatin to cross-link DNA in megakaryocyte progenitors in BM (Zeuner et al., 2007). Our data showed that D-dencichine was unable to promote megakaryocyte polyploidy in BM and spleen cells (**Figures 5A,B**). These data suggested that D-dencichine was unable to promote megakaryocyte proliferation, differentiation, and polyploidy (**Figures 5A–D**).

### D-dencichine Is a Positive Regulator of TPO-Dependent Signaling

**FIGURE 6:**
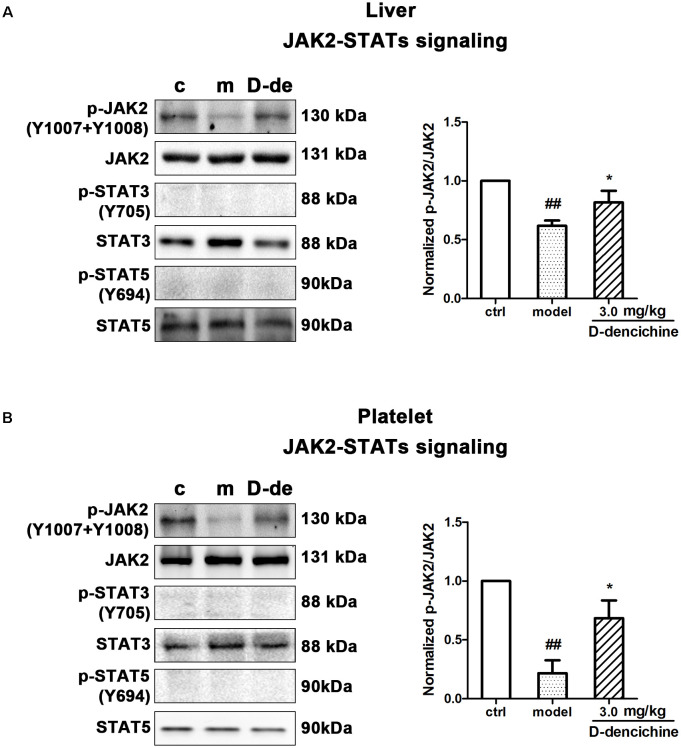
Effects of D-dencichine treatment on JAK2-STATs signaling in mouse liver and platelets. **(A,B)** The expression of p-JAK2/JAK2, p-STAT3/STAT3, p-STAT5/STAT5 proteins in liver and platelet were determined by western blot. Quantification analysis of p-JAK2/JAK2 proteins from the information in corresponding protein bands. ^##^*P* < 0.01 vs. control group; ^∗^*P* < 0.05 vs. model group.

**FIGURE 7:**
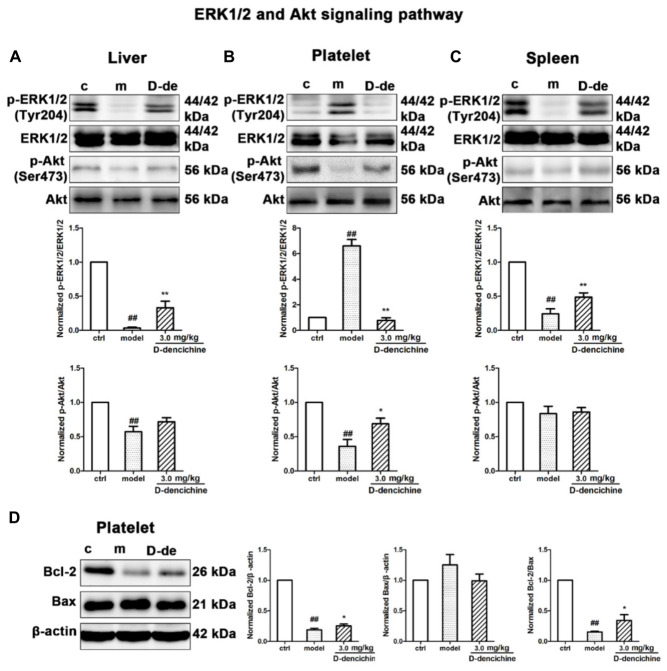
Thrombopoietin-dependent signaling is enhanced with D-dencichine treatment in mouse liver, platelet, and spleen. **(A–C)** Western blots analysis of p-ERK1/2/ERK1/2, p-Akt/Akt proteins in liver, platelet, and spleen. Quantification analysis of TPO-dependent signaling proteins from the information in corresponding protein bands. **(D)** Effects of apoptosis-related protein expression with D-dencichine treatment in mouse platelet. The level of Bcl-2/Bax was determined by western blot. Quantification analysis of Bcl-2, Bax and Bcl-2/Bax expression ratio from the information in corresponding protein bands. ^##^*P* < 0.01 vs. control group; ^∗^*P* < 0.05, ^∗∗^*P* < 0.01 vs. model group.

Elevated platelet numbers in murine models of carboplatin-induced thrombocytopenia suggested that JAK2-STATs and/or TPO-dependent signaling might be involved in D-dencichine treatment of thrombocytopenia. Based on the TPO lever that was dramatically altered in D-dencichine treatment groups, we concluded that appropriate signaling pathways may become active in hepatocytes. Hence, we determined the known cellular signal pathways of JAK2-STATs (Majka et al., 2002) and TPO-dependent signaling, including ERK1/2 and Akt, which are two key regulators (Nakao et al., 2008; Mazharian et al., 2009). In liver and platelet cells, protein investigation revealed that D-dencichine treatment increased the phosphorylation of JAK2 compared with that in the model group (**Figures 6A,B**). In contrast, the phosphorylation states of STAT3 and STAT5 (downstream effectors of JAK2 signaling) were not altered. The data suggested that D-dencichine was capable of increasing the phosphorylation of JAK2 but not completely facilitating the JAK2-STATs signaling pathways. In addition to the liver and platelets, spleen signaling proteins should be paid attention to. On the other hand, we examined whether key regulators of TPO-dependent signaling were altered in liver, platelet and spleen cells with D-dencichine treatment. The examination (**Figures 7A–C**) showed that D-dencichine treatment was capable of increasing the phosphorylation of Akt in platelet cells compared with that in the model group. The increase in Akt phosphorylation was not significant in the liver of D-dencichine treatment and model groups, whereas Akt phosphorylation was unaltered in spleen of each group. Similarly, we found that ERK1/2 phosphorylation in D-dencichine treatment group was enhanced compared with that in the model group in terms of the liver and spleen. Increase in Akt phosphorylation was significant, but the increase in ERK1/2 phosphorylation was more significant. Intriguingly, ERK1/2 phosphorylation was significantly attenuated in D-dencichine treatment group compared to the model group, which was in contrary to the results in liver and spleen samples.

Platelets undergo a specialized form of apoptosis to prolong its survival (Debrincat et al., 2015). The opposite result of ERK1/2 phosphorylation could be related with the platelet survival or apoptosis pathway (Katz et al., 2009; Zhang et al., 2011). Platelet apoptosis is triggered by pharmacological inhibition or conditional loss of Bcl-xL; in addition to thrombocytopenia, a small but significant decrease existed in Bak/Bax (Lebois and Josefsson, 2016). To further reveal the underlying mechanisms, we investigated the effects of D-dencichine treatment on the activation of Bcl-2 and Bax, which regulate the intrinsic pathway to apoptosis (Youle and Strasser, 2008). Western blot analysis showed that D-dencichine treatment caused a stronger activation of Bcl-2/Bax compared with that in the model group (**Figure 7D**).

### D-dencichine Significantly Stimulates Adhesion, Migration, and PPF of Megakaryocytes Through the ERK1/2 and Akt Signaling Pathways

**FIGURE 8:**
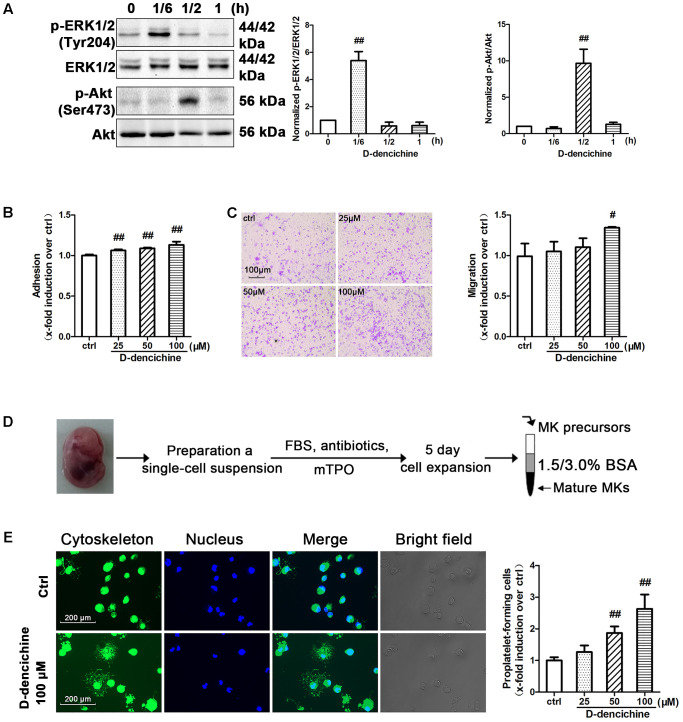
D-dencichine promotes the adherence, migration and PPF of megakaryocytes. **(A)** Western blot analysis of p-ERK1/2 and p-Akt in whole-cell lysates from Meg-01 cells after exposure to D-dencichine (100 μM) for the indicated times after starvation. Quantification analysis of p-ERK1/2/ERK1/2 and p-Akt/Akt from the information in corresponding protein bands. **(B)** Adherent Meg-01 treated with different concentrations of D-dencichine (25, 50, and 100 μM). **(C)** Representative images of migrated megakaryocytes after D-dencichine stimulation. Histogram of migrated megakaryocytes treated with different concentrations of D-dencichine. **(D)** The experimental process of fetal liver-derived megakaryocytes culture and purification. **(E)** Representative photographs of PPF after D-dencichine stimulation. Cytoskeleton actin (green) and the nucleus (blue) were stained. Quantification of PPF megakaryocytes treated with D-dencichine at different concentrations under an inverted microscope. ^#^*P* < 0.05, ^##^*P* < 0.01 vs. control group.

D-dencichine treatment displayed a strong effect on the activation of ERK1/2 and Akt in Meg-01 cells, and their phosphorylation states were rapid and transient (**Figure 8A**). The results strongly revealed that D-dencichine was a positive regulator of TPO-dependent signaling, especially in increasing ERK1/2 phosphorylation. Western blot results indicated that D-dencichine might exert its effects in the terminal stage of thrombocytopoiesis. This process is highly dependent upon a complex network of protein filaments that represent the molecular struts and girders of the cell. Tubulin and actin are both major components of this cytoskeletal network (Italiano et al., 1999). Megakaryocytes migration from a proliferative osteoblastic niche within the BM environment to a capillary-rich vascular niche is an important step for platelet production. Cellular behaviors within adhesion, migration, and PPF, are crucial for megakaryocyte maturation and platelet production (Avecilla et al., 2004). Therefore, we performed mature Meg-01 cells to attach to fibrinogen for 3 h, and the number of attached cells was determined. The attachment to fibrinogen was significantly increased with D-dencichine treatment in a dose-dependent manner compared with that in the control group (**Figure 8B**). Similarly, we also found that D-dencichine had a strong ability to promote megakaryocyte migration compared with that in the control group (**Figure 8C**). We then chose fetal liver-derived primary megakaryocytes to investigate the effects of D-dencichine stimulation on PPF by immunofluorescence. Fetal liver-derived primary megakaryocyte cells were obtained from whole livers recovered from mouse fetuses between embryonic days 13 and 15. As shown in **Figure 8D**, after 5 days of culture in the presence of rmTPO, cell population was purified and enriched on mature megakaryocytes using a 2-step of 1.5%/3% BSA density gradient under gravity (1g) for 45 min at RT. The proportion of mature megakaryocytes in the enriched population was estimated to >60% (data not shown). Afterwards, we analyzed PPF in D-dencichine-stimulated fetal liver-derived primary megakaryocytes after their attachment to immobilized fibrinogen. We found that D-dencichine treatment distinctly promoted PPF characterized by generating large pseudopod-like structures that were elongated, thin, and branched to yield slender tubular projections in a dose-dependent manner compared with that in the control group (**Figure 8E**). This result suggested that D-dencichine regulated PPF, and the further analysis could be done to investigate this complicated process. Taken together, these results indicated that D-dencichine treatment could enhance megakaryocyte adhesion, migration, and PPF through ERK1/2 and Akt pathways.

## Discussion

Patients who have received chemotherapy for supportive treatment of malignancy may develop severe thrombocytopenia that is potentially life-threatening because of the risk of uncontrollable hemorrhage. The list of antitumor drugs is too extensive to be dealt with in the clinical setting. Although drug-induced thrombocytopenia frequently occurs in patients with leukemia, this condition is also observed in patients with solid tumors and associated with increased morbidity and occasional mortality (Zeuner et al., 2007). The scientific research on development of novel thrombopoietic agents for the treatment of patients with thrombocytopenia has attracted an increasing amount of attention because CIT is a predominant clinical abnormality. Carboplatin, highly effective in treatment of malignant tumors, is a well-documented cause of thrombocytopenia in patients (Smith et al., 1993; Ulich et al., 1995). The present study demonstrates that D-dencichine fulfills the promise of ameliorating thrombocytopenia in a mouse model induced by high-dose carboplatin.

Dencichine is a non-protein amino acid that was originally extracted from *Panax notoginseng* (Burk) F. H. Chen and can be artificially synthesized. However, ingestion of *Lathyrus sativus* (grass pea seeds) rich in dencichine results in progressive neurodegenerative symptoms, such as astasia, head retraction, neck stiffness, and extensor paralysis of the legs (Pratap Rudra et al., 2004; Huang et al., 2014), indicating that dencichine has neurotoxic potential. The neurotoxic effects of dencichine have also been demonstrated in several animal species such as mice (Olney et al., 1976) and monkeys (Rao et al., 1971) following systemic administration. An increasing body of evidence has suggested that dencichine is an excitatory acid and acts as an agonist of certain glutamate receptors (Pearson and Nunn, 1981; Ross et al., 1989). D-dencichine, the dextro isomer of dencichine, can be artificially synthesized in the laboratory. Unlike the naturally occurring neurotoxin of dencichine, D-dencichine does not produce any neurological or visible toxic symptoms when administrated at a high dose in animals. SLN Rao et al. (1971) have demonstrated that D-dencichine failed to produce neurotoxicity when administered intraperitoneally even at a high dose of 11 mg/g in day-old chicks, whereas dencichine produced typical neurological symptoms at a dosage of 0.22 mg/g. Bridges et al. (1991) reported that 2 mM initial concentration of dencichine produced glial lysis while the same dosage of D-dencichine had no such effect. Since D-dencichine is not neurotoxic, it implies that the mechanism of toxicity of dencichine is the result of a stereospecific interaction. To our knowledge, the study is the first to show that D-dencichine had significant action on thrombopoiesis. We found that sequential administration of D-dencichine enhanced the production of newly formed platelets. Increased platelet count in the peripheral circulation, combined with its elevated production and its normal clearance, suggested that the effects of D-dencichine on platelet production may be the result of the production of newly platelets.

Platelet production is a consecutive process that starts with megakaryocyte production and ends with the fragmentation of megakaryocytes. Megakaryocytes proliferation and maturation are modulated by multiple growth factors and chemokines. TPO, acting by binding to a specific cell surface receptor (c-Mpl), is the principal hematopoietic cytokine that regulates platelet production and increases survival in animal models of CIT. Contrary to what was expected, serum TPO concentrations in D-dencichine treatment group were lower than those in the model group. The opposite results could be interpreted by the serum TPO being rapidly adsorbed and internalized by receptor c-Mpl in platelets; thus, the platelet release is regulated in part by platelet consumption (Nga et al., 2014). Our data were consistent with previous reports that a feedback loop mechanism between platelet count in the blood and serum TPO levels indeed exists (Emmons et al., 1996). The *in vivo* effects on platelet production of several growth factors other than TPO have been previously studied. For example, IL-6 is another important growth factor and cytokine *in vivo* that acts at multiple steps to elevate platelet counts. IL-6 has been reported to increase the platelet counts in carboplatin-pretreated rats (Mizushima et al., 1992). IL-6 is also reported to exhibit the ability to act directly on megakaryocytes to increase platelet counts (Burstein, 1994; Kaser et al., 2001), and act on hepatocytes to increase the production and release of TPO (Heits et al., 1999). Surprisingly, serum IL-6 levers showed a similar decreasing trend as TPO. The variation trend of IL-6 can be illustrated by its bidirectional function. IL-6 is not as specific growth factor for platelet production as is TPO. Hill et al. (1992) showed that IL-6 levels did not increase after the induction of acute and severe thrombocytopenia. The evidence strongly demonstrated that IL-6 did not mediate thrombopoietic response to acute thrombocytopenia. Although prolonged administration of IL-6 has been shown to induce thrombocytosis, IL-6 and TPO are apparently different and immunologically distinct molecules. On the other hand, IL-6 apparently plays a prominent role in inflammatory and neoplastic diseases. The observations of decreased levels of IL-6 in D-dencichine treatment groups compared with that in model group suggested that IL-6 did not play a role in the stimulation of platelet production in certain inflammatory states of carboplatin-induced myelosuppressed in this study. These findings revealed that D-dencichine was able to play a dramatically regulatory role in control of TPO and IL-6 returning to normal values.

Thrombopoiesis involves several consecutive stages, and its early and middle stages include the commitment of multipotent HSCs toward megakaryocyte progenitors, their proliferation, differentiation, and polyploidization (Chen et al., 2013). Therefore, we focused our initial studies on its early and middle stages. However, the results demonstrated that D-dencichine treatment could not promote the proliferation in both M07e and Meg-01 cells. The aforementioned results urged us to investigate megakaryocyte differentiation with D-dencichine treatment. The expression of CD41α^+^CD42b^+^ in human primary megakaryocytes began to increase at day 7, reached a relatively higher level at day 10, and an even higher level at day 13, but the increase was not significant in D-dencichine treatment groups and control group. The data revealed that D-dencichine had inability to promote the differentiation of cord blood-derived megakaryocytes. Furthermore, as shown in **Figures 2A,C**, we found that the numbers of megakaryocytes in BM were higher in D-dencichine treatment groups than that in the model group, but the increases were not significant. Our findings suggested that D-dencichine had no direct effect on the early and middle stages of thrombopoiesis.

To further verity these data and search for the underlying mechanisms of D-dencichine action on thrombopoiesis, we investigated the related signaling pathways. Previous studies have revealed that JAK2-STAT3/5 signaling is involved in process of megakaryocytopoiesis and platelet production (Drachman et al., 1995). Inagaki et al. (2004) reported that a novel small molecule (JTZ-132) induces the growth and differentiation of megakaryocytic progenitor cells and improves thrombocytopenia in myelosuppressed mice via tyrosine phosphorylation of c-Mpl, JAK2, and STAT5. A study by Kirito et al. (2002) revealed that STAT3 plays an important role in megakaryopoiesis through the expansion of megakaryocyte progenitor cells. Similarly, Grozovsky et al. (2015) demonstrated that Ashwell-Morell receptor regulates hepatic TPO production via JAK2-STAT3 signaling. However, we observed that D-dencichine treatment could induce the activation of JAK2 phosphorylation, but did not facilitated STAT3/5 phosphorylation. The observation of enhanced phosphorylation of JAK2 indicated that TPO-dependent signaling might be partially activated with the treatment of D-dencichine. These results were consistent with previous reports that JAK2-STATs signaling contributes to megakaryocyte proliferation and differentiation. In addition to JAK2-STAT3/5 signaling involved in thrombopoiesis, the known cellular ERK1/2 and AKT signaling pathways regulate platelet production.

Thrombopoietin-dependent signaling includes two important positive regulators, ERK1/2 and Akt. In our experiments, we found that D-dencichine treatment could induce a rapid and transient phosphorylation of ERK1/2 and slightly delayed the activation of AKT in Meg-01 cells, suggesting that D-dencichine treatment might play a role in the terminal stages of thrombopoiesis. ERK1/2 is an important significant cellular signal transduction pathway involved in the adhesion and migration of megakaryocytes (Mazharian et al., 2009; Chen et al., 2016). Chen et al. (2016) had reported that sympathetic stimulation facilitates thrombopoiesis by promoting megakaryocyte adhesion, migration, and PPF through ERK1/2 signaling. The Akt pathway is known to be crucial for the regulation of megakaryocyte maturation and platelet release, which occur in healthy conditions (Severin et al., 2010). A study by Xu et al. (2014) revealed that dTMP-growth hormone fusion protein promotes megakaryocyte differentiation and PPF through Akt signaling pathway. Investigation of the effect of D-dencichine on the ERK1/2 and Akt signaling pathway further confirmed that D-dencichine played a key role in the terminal stages of thrombopoiesis. On the basis of our findings, we examined D-dencichine treatment in the terminal stages of thrombopoiesis, including adhesion and migration of mature megakaryocytes, extension of proplatelet elongations into the sinusoidal blood vessels, proplatelets continuing to mature in the vasculature, and ultimately releasing individual platelets from their tips (Yin and Li, 2006). We demonstrated that D-dencichine could enhance megakaryocyte adhesion, migration, and PPF in a dose-dependent manner through ERK1/2 and AKT signaling. These results revealed that D-dencichine treatment caused a rapid increase in platelet production by regulating the terminal stage of thrombopoiesis. These findings could explain our observation that the platelet counts of the mice in the D-dencichine treatment group were higher than those of the model group. We further confirmed that ERK1/2 pathway was the key mediator in D-dencichine-induced megakaryocyte adhesion and migration, which was consistent with the previous reports that ERK1/2 signaling is involved in the adherence and mobility of various cell types (Chen et al., 2016). Moreover, D-dencichine treatment could stimulate the activation of AKT, which may result in the expansion of fully differentiated megakaryocytes with an enhanced ability to extend long branched proplatelets. The further analysis should be done to investigate the signaling pathways that facilitated these effects.

Although the mechanisms of the D-dencichine treatment on thrombopoiesis are quite different from those of growth factors, we expect that the combined application of D-dencichine with other kinds of hematopoietic growth factors may achieve the quick recovery of platelets in patients with thrombocytopenia. Collectively, this study sheds new light on the functional role of D-dencichine in promoting thrombopoiesis and provides material for further studies for treating thrombocytopenia.

## Conclusion

Our investigation results represent the first evidence that D-dencichine significantly increases platelet count in the murine model of carboplatin-induced thrombocytopenia, which is associated with its stimulating megakaryocyte adhesion, migration, and PPF through ERK1/2 and Akt signaling pathway. If these effects of D-dencichine are validated in clinical trials, it might be a promising avenue for CIT treating.

## Author Contributions

SD, MW, FW, GS, and XS contributed to design the experiments in the study. SD, MW, and HX took part in the experiments. SD, MW, and HF contributed to analyze the data and revise the manuscript. MW, XQ, and HF designed the protocol for flow cytometry analysis. SF and YT took part in the synthesis of D-dencichine. MW, SL, and YZ helped to perform the analysis with constructive discussions and complete statistical analyses.

## Conflict of Interest Statement

The authors declare that the research was conducted in the absence of any commercial or financial relationships that could be construed as a potential conflict of interest.
