# Corrigendum: D-dencichine regulates thrombopoiesis by promoting megakaryocyte adhesion, migration and proplatelet formation

**DOI:** 10.3389/fphar.2024.1504076

**Published:** 2025-01-23

**Authors:** Shilan Ding, Min Wang, Song Fang, Huibo Xu, Huiting Fan, Yu Tian, Yadong Zhai, Shan Lu, Xin Qi, Fei Wei, Guibo Sun, Xiaobo Sun

**Affiliations:** ^1^ Beijing Key Laboratory of Innovative Drug Discovery of Traditional Chinese Medicine (Natural Medicine) and Translational Medicine, Institute of Medicinal Plant Development, Peking Union Medical College and Chinese Academy of Medical Sciences, Beijing, China; ^2^ Key Laboratory of Bioactive Substances and Resource Utilization of Chinese Herbal Medicine, Ministry of Education, Beijing, China; ^3^ Zhongguancun Open Laboratory of the Research and Development of Natural Medicine and Health Products, Beijing, China; ^4^ Key Laboratory of Efficacy Evaluation of Chinese Medicine Against Glycolipid Metabolic Disorders, State Administration of Traditional Chinese Medicine, Beijing, China; ^5^ Kunming Shenghuo Pharmaceutical Group Co., Ltd., Kunming, China; ^6^ Academy of Chinese Medical Sciences of Jilin Province, Jilin, China; ^7^ Department of Oncology, Guang’anmen Hospital, China Academy of Chinese Medical Sciences, Beijing, China

**Keywords:** chemotherapy-induced thrombocytopenia, thrombopoietin, D-dencichine, platelets, cytokines, mouse

In the published article, there was an error in Figure 7 as published. In Figure 7, the Akt protein bands in the liver group and platelet group were the same in this study. The corrected Figure 7 and its caption, appear below.

**FIGURE 7:**
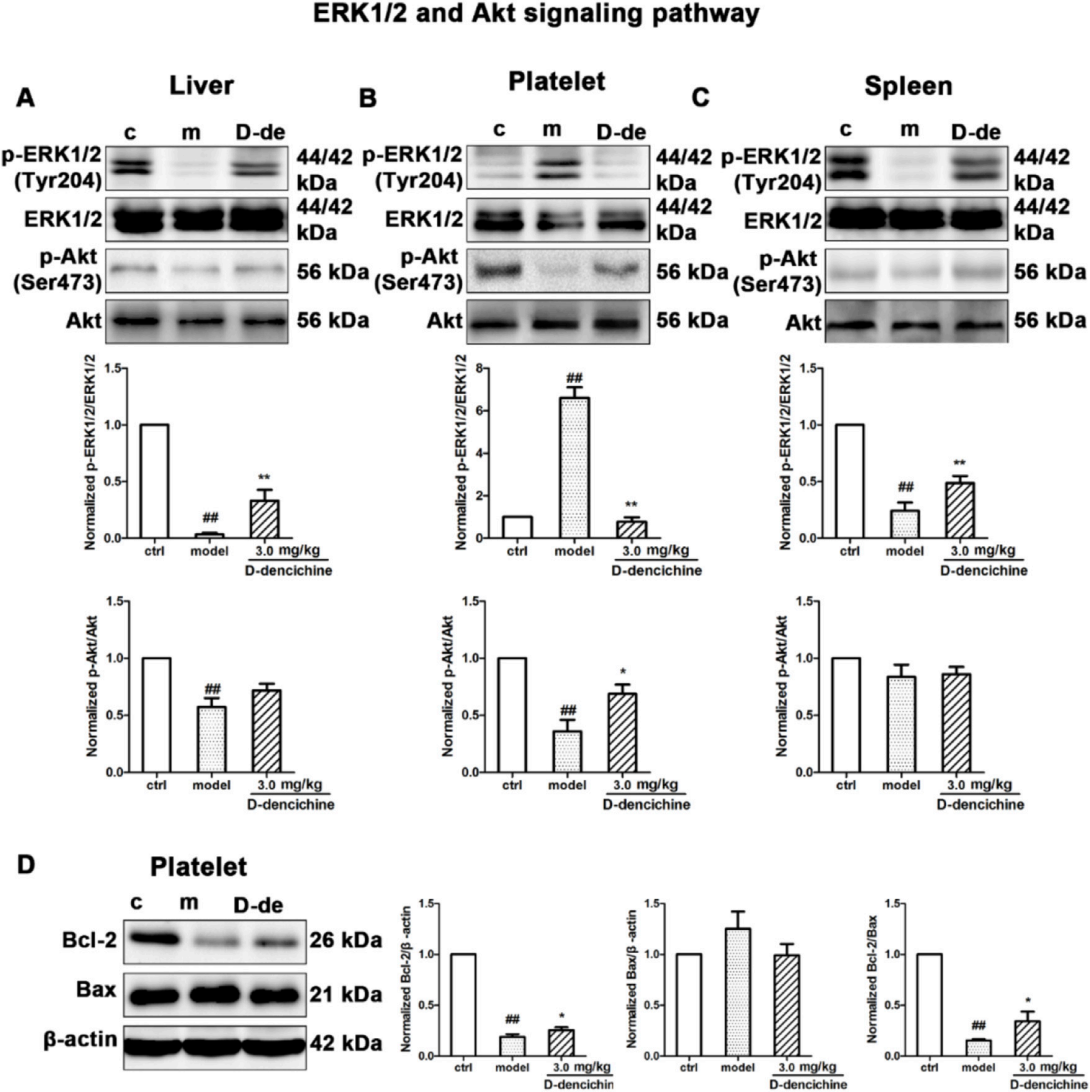
Thrombopoietin-dependent signaling is enhanced with D-dencichine treatment in mouse liver, platelet, and spleen. **(A–C)** Western blots analysis of p-ERK1/2/ERK1/2, p-Akt/Akt proteins in liver, platelet, and spleen. Quantification analysis of TPO-dependent signaling proteins from the information in corresponding protein bands. **(D)** Effects of apoptosis-related protein expression with D-dencichine treatment in mouse platelet. The level of Bcl-2/Bax was determined by western blot. Quantification analysis of Bcl-2, Bax and Bcl-2/Bax expression ratio from the information in corresponding protein bands. ^##^ *P* < 0.01 vs. control group; ^*^ *P* < 0.05, ^**^ *P* < 0.01 vs. model group.

The authors apologize for this error and state that this does not change the scientific conclusions of the article in any way. The original article has been updated.

